# Lipid Membrane Mimetics in Functional and Structural Studies of Integral Membrane Proteins

**DOI:** 10.3390/membranes11090685

**Published:** 2021-09-03

**Authors:** Saman Majeed, Akram Bani Ahmad, Ujala Sehar, Elka R. Georgieva

**Affiliations:** 1 Department of Chemistry and Biochemistry, Texas Tech University, Lubbock, TX 79409, USA; saman.majeed@ttu.edu (S.M.); abaniahm@ttu.edu (A.B.A.); usehar@ttu.edu (U.S.); 2 Department of Cell Physiology and Molecular Biophysics, Texas Tech University Health Science Center, Lubbock, TX 79409, USA

**Keywords:** integral membrane proteins, lipid membrane mimetics, detergent micelles, bicelles, nanodiscs, liposomes

## Abstract

Integral membrane proteins (IMPs) fulfill important physiological functions by providing cell–environment, cell–cell and virus–host communication; nutrients intake; export of toxic compounds out of cells; and more. However, some IMPs have obliterated functions due to polypeptide mutations, modifications in membrane properties and/or other environmental factors—resulting in damaged binding to ligands and the adoption of non-physiological conformations that prevent the protein from returning to its physiological state. Thus, elucidating IMPs’ mechanisms of function and malfunction at the molecular level is important for enhancing our understanding of cell and organism physiology. This understanding also helps pharmaceutical developments for restoring or inhibiting protein activity. To this end, in vitro studies provide invaluable information about IMPs’ structure and the relation between structural dynamics and function. Typically, these studies are conducted on transferred from native membranes to membrane-mimicking nano-platforms (membrane mimetics) purified IMPs. Here, we review the most widely used membrane mimetics in structural and functional studies of IMPs. These membrane mimetics are detergents, liposomes, bicelles, nanodiscs/Lipodisqs, amphipols, and lipidic cubic phases. We also discuss the protocols for IMPs reconstitution in membrane mimetics as well as the applicability of these membrane mimetic-IMP complexes in studies via a variety of biochemical, biophysical, and structural biology techniques.

## 1. Introduction

**Figure 1:**
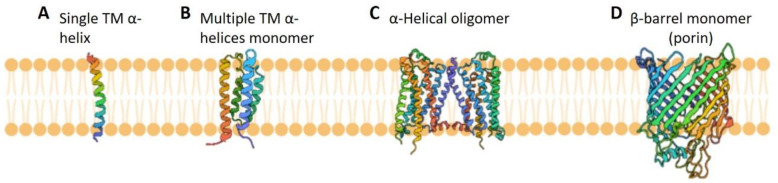
Representative types of IMPs: The α-helical IMPs can have just one helix (**A**) or multiple helices (**B**) that traverse the membrane; they can be multimeric as well (**C**). The β-barrel membrane proteins typically have multiple membrane-traversing strands (**D**) and can be either monomeric or oligomeric. The lipid membrane bilayer is shown in orange. The structures of IMPs with PDB accession codes 5EH6 (**A**), 2KSF (**B**), 5OR1 (**C**), and 4GPO (**D**) are shown in the figure. The membrane orientation was not considered.

Integral membrane proteins (IMPs) (Figure 1) reside and function in the lipid bilayers of plasma or organelle membranes, and some IMPs are located in the envelope of viruses. Thus, these proteins are encoded by organisms from all living kingdoms. In almost all genomes, approximately a quarter of encoded proteins are IMPs [1,2] that play critical roles in maintaining cell physiology as enzymes, transporters, receptors, and more [3,4,5]. However, when modified via point mutations, deletion, or overexpression, these proteins’ function becomes abnormal and often yields difficult- or impossible-to-cure diseases [6,7]. Because of IMPs’ important role in physiology and diseases, obtaining their high-resolution three-dimensional (3D) structure in close to native lipid environments; elucidating their conformational dynamics upon interaction with lipids, substrates, and drugs; and ultimately understanding their functional mechanisms is highly important. Such comprehensive knowledge will greatly improve our understanding of physiological processes in cellular membranes, help us develop methodologies and methods to overcome protein malfunction, and improve the likelihood of designing therapeutics for protein inhibition. Notably, it is remarkable that almost 40% of all FDA-approved drugs exploit IMPs as their molecular targets [8,9].

Undeniably, functional and structural studies of IMPs have greatly advanced in recent decades by developing diverse in-cell and in-vitro functional assays [10,11,12,13]; advancing the X-ray crystallography applications for membrane proteins in detergents [14,15], bicelles, nanodiscs, and lipidic cubic phases [15,16,17,18,19,20] to determine the structure at a typical 3 Å or even higher resolution; improving data detection and processing for single-particle cryo-electron microscopy (cryoEM) to increase the number of resolved IMPs’ structures at ca. 3.5–3 Å resolution [21,22,23]; the contribution from single-molecule FRET spectroscopy (smFRET) toward understanding IMPs’ conformational dynamics in real time under physiological environment conditions [24,25,26]; the growing number of highly sophisticated studies using EPR spectroscopy via continuous wave (CW) and pulse methods to uncover the short- and long-range conformational dynamics underlying IMPs’ functional mechanisms [27,28,29,30,31,32,33]; advancing NMR spectroscopy [34,35,36] and particularly solid-state NMR applied to proteins in lipid-like environments [37,38,39]; conducting extensive studies using site-directed mutagenesis to identify the roles of certain amino acid residues in the IMPs’ function [40,41,42], molecular dynamics computational studies [43,44,45]; and more. Despite this substantial progress, IMPs are still understudied and require further research.

The enormous diversity and complexity of IMPs challenges researchers because they must uncover and characterize numerous diverse functional mechanisms. Any step in the workflow, from gene to characterizing IMPs’ structure and function can present challenges, such as poor solubilization efficiency from the host cell membrane, limited long-term stability, low protein expression, and more [46,47,48]. Another serious issue is identifying and developing appropriate membrane protein hosts, i.e., lipid membrane-like mimetics, to which IMPs are transferred from the native membranes where they are expressed, or from inclusion bodies in the case of eukaryotic or viral proteins produced in *E. coli* [49]. This is needed for further purification and in vitro functional and structural studies [50,51,52,53,54]. In general, IMPs are difficult to solubilize away from their native environment in the cell membrane due to their hydrophobic regions [55]. Also, removing these proteins from their native cellular form sometimes results in evident functional and structural implications [54]. Thus, selecting a suitable membrane mimetic for each particular protein is critical for obtaining samples of functional proteins for in vitro studies on active or purposely inhibited protein states. Furthermore, the isolated and purified IMPs often need to be obtained at concentrations and purity, which are satisfactory for the biochemical and biophysical techniques used for these proteins’ characterization.

Due to the high importance of membrane mimetics for accommodating and maintain IMPs’ native state, special attention must be paid to the current state and further prospective when developing these nano-sized membrane platforms. Therefore, we focus here on reviewing the most widely used and emerging membrane mimetics, which are detergents, multilamellar lipid emulsions, unilamellar liposomes, Lipodisqs^®^/nanodiscs, bicelles, amphipols, and lipidic cubic phases (LCPs), in IMP purification and structure–function studies. Additionally, we describe applications of these mimetics for particular IMPs and discuss how selecting a membrane mimetic affects these proteins’ properties. Of course, due to rapidly increasing contributions in the field and space limitations, this review cannot cover all the developments and applications of membrane mimetic systems and their applications in membrane functional and structural molecular biology studies.

## 2. An Overview of the Most Widely Used Lipid Membrane Mimetics and Their Applications in Functional and Structural Studies of Integral Membrane Proteins

The development of lipid membrane mimetics to make IMPs amenable for isolation, purification, and in vitro characterization has a long history. Generally, a membrane mimetic should reproduce the lipid bilayer properties, or at least recreate the hydrophobic core environment of a lipid bilayer in its most fundamental form [54,56]. Although detergents have been the most widely used substitute for the membrane environment, in the recent decades a great deal of effort has been invested to expand the diversity of membrane mimetics and to use more lipid bilayer-like structures, which together with the incorporated proteins have high solubility and stability. These novel membrane mimetics provide the following advantages for the incorporated IMPs: (i) convenience to investigate them via research technologies that are impossible or difficult to execute in the presence of detergents, (ii) improved stability, and (iii) provision of an environment with a chemical composition and/or physical characteristics closer to the native lipid membrane bilayer environment [57]. However, all of these membrane mimetics have pros and cons, and not all are compatible with various protein research techniques.

Here, we further describe these membrane mimetics and discuss their applications in studying IMPs.

### 2.1. Detergents and Detergent Micelles in Studies of Integral Membrane Proteins

#### 2.1.1. General Properties of Detergents and Detergent Micelles

**Figure 2:**
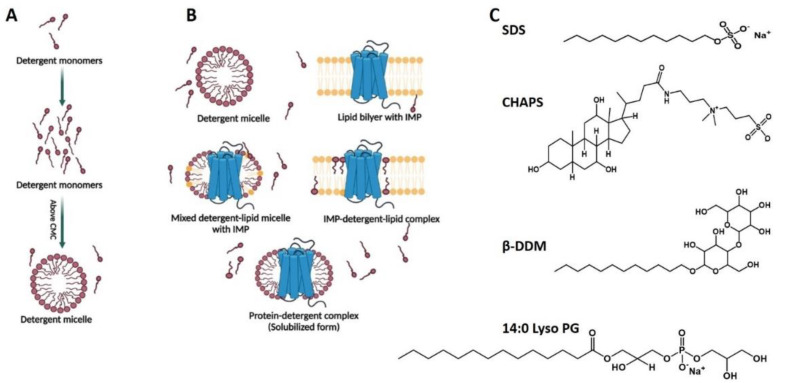
IMPs in detergents: (**A**) In aqueous solution, above a certain concentration (CMC), detergent molecules self-associate to form close to spherical aggregates (micelles) with hydrophilic and hydrophobic portions facing the aqueous environment and the micelle interior, respectively. (**B**) Detergents are used for the extraction of IMPs from the native membrane of expression host—detergent at a high concentration, much above its CMC, is mixed with the native membranes containing the IMP of interest; due to its hydrophobic properties the detergent mixes with the membrane lipids and solubilizes the membrane; as a result, mixed IMP–lipid–detergent, IMP–detergent or detergent–lipid complexes are formed; thereafter, the lipid molecules are removed in the next purification steps unless specific lipids are tidily bound to the IMP. (**C**) The chemical formulas of some of the most widely used in studies of IMPs detergents are shown: SDS is negatively charged, CHAPS is zwitterionic, DDM is non-charged; and 14:0 Lyso PG is negatively charged.

Detergents are the archetypal lipid membrane mimetics and have been extensively utilized for the solubilization and characterization of IMPs. They are amphipathic molecules and, above a certain so-called critical micelle concentration (CMC), self-aggregate to form micelles in aqueous solutions (Figure 2A). Saponins and naturally occurring bile salts were the first detergents used for biochemical studies [58,59]. Currently, ample diverse detergents with variable biochemical and biophysical characteristics are available.

Detergents fit into three major classes (Figure 2C): *ionic detergents* have either positively or negatively charged headgroups and are strong denaturants or harsh membrane mimetics owing to their effect on IMPs’ structure, e.g., sodium dodecyl sulfate (SDS) has negatively charged headgroups; *zwitterionic detergents*, e.g., the traditional 3-[(3-cholamidopropyl)dimethyl-ammonio]-1-propane-sulfonate (CHAPS) or Lauryl-dimethylamine-N-oxide (LDAO), have zero overall molecular charge, exhibit a less pronounced denaturation effect compared to ionic detergents and a stronger solubilization potential compared to non-ionic detergents, and are hence categorized as an intermediate between non-ionic and ionic detergents; and *non-ionic detergents* are comparatively mild, have non-charged hydrophilic groups, tend to shield the inter- and intra-molecular protein–protein interactions and maintain the structural integrity of solubilized proteins, e.g., dodecyl-L-D-maltoside (DDM), lauryl-maltose neopentyl-glycol (LMNG), and octyl-L-D-glucoside (OG) [54,60,61]. Phospholipid-like detergents are either charged, like 14:0 Lyso PG (1-myristoyl-2-hydroxy-sn-glycero-3-phospho-[1′-rac-glycerol]) and 16:0 Lyso PG (1-palmitoyl-2-hydroxy-sn-glycero-3-phospho-[1′-rac-glycerol]), or zwitterionic, like 14:0 Lyso PC (1-myristoyl-2-hydroxy-sn-glycero-3-phosphocholine) and Fos-Choline 12. These have also been extensively used in studies of IMPs [62,63].

#### 2.1.2. Detergent Applications in Integral Membrane Proteins Solubilization, Purification, and Stabilization

Typically, the first step in transmembrane protein purification is extracting it from the host membrane or inclusion body. The protein extraction from the host membrane is carried out by adding an appropriate detergent at a high concentration (several times above the CMC) to the homogenized proteo-lipid membrane, which solubilizes the membrane (Figure 2B). Initially, destabilization and fragmentation of lipid bilayer occur due to inserting the detergent molecules into the membrane. Subsequently, the lipid membrane is dissolved, and then IMP-detergent, lipid-detergent, and lipid-IMP-detergent mixed compositions are formed [64]. Various detergents exhibit different capacities for solubilizing biological membranes. Similarly, the type of detergent used for solubilization can affect the preservation of specifically bound lipid molecules in the IMP’s final detergent-solubilized state [65]. Multiple detergents must be screened to identify those that maintain the IMP’s structural integrity and functional activity, and suit downstream applications [54]. For instance, detergents with a low CMC can effectively solubilize most membranes but are less appropriate for methods requiring detergent removal because they can be difficult to remove later [66]. Also, using a mild detergent that only binds to the transmembrane region of a given IMP and can retain key lipid interactions is essential for successful studies [67]. Once solubilized, the IMPs’ purification follows the same principles as for purifying soluble proteins, utilizing chromatographic methods like affinity, gel filtration, and/or ion-exchange chromatography. Alternatively, when IMPs are deposited into inclusion bodies, such as eukaryotic proteins or prokaryotic outer membrane proteins expressed in *E. coli*, their refolding into detergent micelles is an efficient approach to obtain solubilized membrane proteins in a physiologically-relevant state. Thus, due to their convenience and large variability, detergents are one of the most extensively used membrane mimetics and are almost unavoidably utilized for extracting and solubilizing IMPs from host membranes and for screening for optimal IMP stability [68,69]. In many studies, detergents are also used as intermediate IMP hosts from which the IMP is transferred into more lipid-like and lipid-bilayer-like mimetics, such as nanodiscs, liposomes, and other for additional downstream investigations [54].

On the other hand, the hydrophobic tails of detergent molecules in the micelle, which are shorter and more mobile compared to lipids’ alkyl tails, make an inadequate mimic of the lipid bilayer. Due to a mismatch in hydrophobic thicknesses, the isolated IMPs and the detergent micelle can also influence each other’s shape, leading to the adoption of non-physiological IMP conformations [70]. In addition, the hydrophobic packing in proteo-micelles is weaker than those for IMPs in a lipid bilayer, allowing increased water penetration into the detergent micelle and leading to IMPs’ structural instability [71]. Despite these deficiencies, the detergents and detergent micelles are currently among the most widely used membrane mimetics for in vitro studies of IMPs.

#### 2.1.3. Applications of Detergents in Functional Studies of Integral Membrane Proteins

Although IMPs’ activity assays have been conducted mostly in lipid bilayers and predominantly on liposome-reconstituted IMPs, functional studies of detergent-solubilized IMPs have also been carried out. Studies have investigated substrates’ binding affinities to characterize a critical stage initiating the substrate translocation via membrane transporters and channels. These studies monitored the binding of a radioactively labeled substrate in the case of the prokaryotic Na/tyrosine transporter (Tyt1) [13], and isothermal titration calorimetry (ITC) studies elucidated the binding of ligands (ions and other substrates) to transporter/channel or receptor IMPs [72,73,74,75]. The ATPase activity of ABC transporters in detergents was also examined [76,77]. It was found in such studies that a LmrA transporter in FC-16 detergent has higher ATPase activity and ligand binding compared to LmrA solubilized in DDM [78].

#### 2.1.4. Detergent Applications in Studies of Integral Membrane Proteins Using Biophysical and Structural Biology Methods

Detergent-solubilized IMPs have been extensively studied by almost all available biophysical and structural biology techniques to determine physiologically relevant or disease-linked protein conformations and conformational transitions with and without ligands, e.g., substrates or inhibitors, bound to the protein molecules. Currently, most existing atomic-resolution X-ray crystal structures are of detergent-solubilized IMPs. Importantly, IMPs’ proper folding and monodispersity are critical for a successful crystallization. Several approaches have been utilized to assess the IMP homogeneity: size exclusion chromatography (SEC) with light scattering and sedimentation equilibrium centrifugation analyses [79], fluorescence-detection SEC [80], polypeptide thermal stability using a thiol-specific fluorescent reporter to monitor cysteine residue accessibility upon denaturation [81], *nano*DSF with light scattering [82], and thermal or chemical denaturation using circular dichroism (CD) spectroscopy to monitor the stability of IMPs’ secondary structure [83,84]. Thus, multiple detergents must be screened, and those that maintain protein homogeneity and integrity are considered for further use [82,85]. Still, other factors appear key to successful IMP crystallization. Given that not just the protein, but the protein–detergent complex must crystallize [86], several analyses searched for a trend in the conditions used for obtaining high-quality IMP crystals [87]. Regarding the detergent used, statistics as of 2015 show that half of IMP crystal structures were obtained in alkyl maltopyranosides, followed by the alkyl glucopyranosides (23%), amine oxides (7%), and polyoxyethylene glycols (7%) [87]. The most successful alkyl maltopyranoside detergent is n-dodecyl-β-D-maltopyranoside (DDM), followed by n-decyl-β-D-maltopyranoside (DM) [87]. Thus, in addition to maintaining protein stability, detergents with shorter chain provide a good environment for IMP crystallization because they form smaller micelles, which facilitate tighter packing in the crystal lattice and higher-quality crystal diffraction [82,88,89,90]. The IMP structures from diverse families have been solved, and some of these structures capture the same protein in distinct conformations. This information is invaluable for elucidating functional and/or inhibition mechanisms. IMPs crystallized in detergent include glutamate receptor GluA2 [91], neurotransmitter transporter homologue LeuT [92,93], betaine transporter BetP [94], and many more. The protein data bank (PDB) provides detailed information about IMPs’ deposited crystal structures in detergents.

In the last decade, EM and single-particle cryoEM in particular have made historic progress in studying detergent-solubilized IMPs by expanding this technique’s applications to diverse families of IMPs and by determining these proteins’ 3D structure at high resolution down to ca. 3 Å [21,95]. In contrast to X-ray crystallography, EM does not require protein-crystal formation and has much more potential to deal with conformationally heterogeneous proteins and protein complexes. Nevertheless, successful IMP structure determination via EM requires high stability and proper folding of the detergent-solubilized protein [95]. For this reason, detergents are screened similarly to the crystallization of IMPs. In addition, EM sometimes experiences specific problems with detergents suitable for crystallization, including the detergents DDM or LMNG. It can be difficult to distinguish the protein particle from a detergent via a negative EM stain, as found in the study of citrate transporter CitS in DDM and DM [96]. To reduce the background and facilitate visualizing protein particles, free detergent micelles can be removed prior to the EM experiments [97]. In contrast, other studies found that detergents with low CMC, such as DDM and maltose-neopentyl glycols (MNGs), provide a better platform for a single-particle cryoEM of IMPs [98]. Another detergent used in cryoEM structure determination is digitonin (an amphipathic steroidal saponin) [99]. Fluorinated Fos-Choline-8 detergent was also used to stabilize and determine the structure of a homo-oligomeric serotonin receptor in its apo, serotonin-bound, and drug-bound states [100,101,102].

Solution NMR spectroscopy has also benefited from detergent-solubilization in studying the high-resolution structure of full-length (FL) IMPs or truncated IMP constructs and in monitoring the conformational transitions in IMPs’ monomers and complexes [103]. Specifically for NMR, despite the significant technical and methodological advancements in recent decades, this method is still limited by the protein’s size; in the case of IMPs, this includes the size of a membrane mimetic-protein complex. Thus, the slow tumbling of large-protein objects in a solution significantly shortens the traverse relaxation times resulting in NMR line broadening, and ultimately causes a loss of NMR sensitivity [103]. The large size of protein molecules also produces overcrowded NMR spectra, which are difficult to interpret. Therefore, the current size limit for proteins and protein complexes studied by NMR in solution does not exceed 70 kDa even when advantageous pulse sequences are applied [103,104,105]. Given this, solution NMR studies on IMPs require detergent micelles to be as compact (small) as possible but still adequately mimic the membrane environment [103]. Care must be taken to achieve high monodispersity of the studied IMP. The length of IMP transmembrane segments should also generally match the micelle hydrophobic core to avoid inconsistent NMR data [106]. Historically, “harsh” detergents like dodecylphosphocholine (DPC) and lauryldimethylamine-N-oxide (LDAO) that form small micelles (20–25 kDa) and maintain IMPs functional states have been used to study the human VDAC-1 [107], the human voltage-dependent anion channel [108], the outer membrane protein G [109], and more. Mild detergents, like DM and DDM have been used in NMR solution studies of bacteriorhodopsin [110], G-protein-coupled receptors (GPCRs) [111,112], voltage-dependent K^+^ channels [113], and more. IMPs solubilized in micelles of anionic lysolipids (e.g., 14:0 PG and 1-palmitoyl-sn-glycero-3-phospoglycerol [16:0 PG]) and short-chain lipids (e.g., 1,2-dihexanoyl-sn-glycero-3-phosphocholine [DHPC]) have been studied by NMR in solution [114,115,116,117].

EPR spectroscopy, continuous wave (CW), and pulse, in combination with spin labeling [27,30,31,118,119,120,121,122,123], have provided invaluable information about the conformational dynamics and function/inhibition of IMPs. These studies were conducted exclusively or partly on detergent-solubilized IMPs. Large structural rearrangements in DDM–solubilized membrane transporters, which report on protein dynamics along the transport cycle or the assembly into functional units, were uniquely captured by pulse EPR distance measurements [28,32,124,125,126,127,128,129,130,131]. Viral, bacterial, and eukaryotic channels [29,132,133], receptors [134,135], and more were also studied in detergent micelles (DDM, DM, lauryl maltose neopentyl glycol [MNG], etc.) via CW and pulse EPR spectroscopy. Importantly, EPR spectroscopy experiments have no specific requirements for the detergent used insofar as the detergent supports protein stability. Also, there is no restriction on IMP’s size, given that the protein can be successfully spin-labeled. Moreover, EPR spectroscopy can investigate IMPs within a broad range of concentrations (e.g., ca. <5 µM to >100 µM), allowing researchers to capture multimeric IMP intermediates in detergent micelles [29].

Another informative technique in studies of detergent-residing IMPs is fluorescence spectroscopy/microscopy, exemplified by Förster resonance energy transfer (FRET) spectroscopy and particularly by the single-molecule FRET (smFRET) version [136]. It captures conformational motions within one protein molecule/complex in real time, although measurements on many molecules/complexes are needed to average the effect of modulating protein conformation by, for example, ligand binding. smFRET has been used in multiple studies on detergent-residing IMPs to monitor their conformational responses to ligands, changes in pH, or other stimuli [137,138,139].

### 2.2. Bicelles in Studies of Integral Membrane Proteins

#### 2.2.1. General Properties of Bicelles

**Figure 3:**
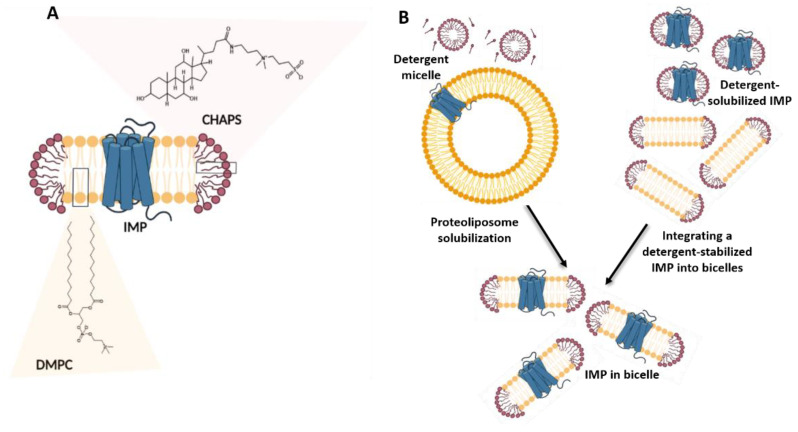
IMPs in bicelles. (**A**) Bicelle-residing IMP containing multiple transmembrane helices is shown; the bicelle is composed of a patch of bilayer-forming lipids (e.g., DMPC) stabilized by short-chain lipid or detergent (e.g., CHAPS). The size of bicelles depends on the molar ratio between long- and short-chain lipids used in their preparation (Equation (1)). In addition, bicelle size is affected also upon dilution of the bicellar solution. (**B**) Two major protocols for incorporation of IMPs into bicelles are outlined: detergent/detergent micelles are mixed with proteoliposomes (**left**) or IMP in detergent micelles are mixed with lipids and bicelle-forming detergent (**right**). The figure shows simplified procedures.

Introduced by Prestegard and colleagues in 1988, bicelles (binary/bi-layered mixed micelles) are recognized as the first lipid membrane mimetic system capable of incorporating a substantial amount of lipids to create a bilayer-like environment for membrane proteins [140]. Bicelles are disc-shaped nanoaggregates comprising bilayer-forming long-chain phospholipids mixed with either detergent molecules or short-chain phospholipids in an aqueous environment [69,140] (Figure 3A). They are an attractive membrane mimetic for studying the structure and structural dynamics of membrane proteins. For example, isotropic bicelles can be formed in aqueous solutions by mixing the long-chain lipid 1,2-dimyristoyl-sn-glycero-3-phosphocholine (DMPC) with the detergent 3-[(3-cholamidopropyl)dimethyl-ammonio]-1-propane sulfonate (CHAPS). They can also be formed by mixing the long-chain lipids 1,2-dimyristoil-sn-Glycero-3-[Phospho-rac-(1-glycerol)] (DMPG) and DMPC with the short-chain lipid DHPC [141,142]. Bicellar nanostructures comprising various lipids with incorporated cholesterol, ceramides, cardiolipin, and more have also been developed [143,144,145].

Generally, geometric arguments can help to estimate the bicelle’s size using the molar ratio between long- and short-chain lipids (or detergent); this so-called *q* value (Equation (1)) to calculate the radius of the bicelle’s bilayer region (R) directly, in addition to the bicelle’s topology and size [146,147,148].

(1) $$q=\frac{total\;molarirty\;of\;long-chain\;lipid}{total\;molarity\;of\;detergent\;\left(short-chain\;lipid\right)-CMC\;of\;detergent\;\left(short-chain\;lipid\right)}\;$$

In addition, dynamic light scattering and NMR can also be used to experimentally determine bicelles’ size and morphology in an aqueous buffer at a constant total lipid/detergent concentration [149,150].

Bicelles with a higher *q* value are formed from low concentrations of short-chain lipids/detergents in relation to the concentration of long-chain lipids, and they are typically larger than the low *q*-value bicelles. Bicelles with smaller *q* values (*q* ≤ 0.6) are more “detergent-rich” and “lipid-poor”, so the phospholipid environment they provide can perturb the bicelle-incorporated IMP [146]. However, it is difficult to precisely estimate bicelle size. For example, bicelles made of DMPC/DHPC had an estimated average size of 20 nm at *q* = 2 [143], and those made of DMPC/DMPG/DHPC at *q* = 2.6 had an estimated average size of 10 nm [149]. This discrepancy can be explained by the limitations of different methods used to determine bicelles’ size. IMPs have been reconstituted and studied in both large and small bicelles [146,147].

Due to bicelles’ small size, their suspensions are effectively homogeneous and translucent even after incorporating membrane proteins [151,152]. One major advantage of this membrane mimetic system is its resemblance to a small fragment of lipid bilayer. In addition, embedding IMPs in a native-like environment and a simple variation in the *q* value can help in the system’s size scalability [153]. Furthermore, native bicelles made of lysed eukaryotic-cell lipids mixed with DHPC were also prepared to provide diverse lipid types for specific interactions with proteins [154]. Thus, bicelles outperform detergents in maintaining membrane proteins’ functional state. In addition, paramagnetic ions can be added to the lipid mixtures, so the resulting bicelles can align in an external magnetic field, aiding magnetic resonance studies on IMPs [155,156].

Notably, the presence of detergent-like short-chain lipids and a bilayer size is insufficient to provide membrane-like lateral pressure and may perturb the structure and dynamics of bicelle-residing IMPs [54,69,157]. Another disadvantage of conventional bicelles is that their size and geometry depend on the total lipid concentration in the solution; therefore, any dilution changes the system properties. At high dilutions, bicelle-to-vesicle transitions can occur [143], so care must be taken to maintain constant lipid concertation throughout the experiment. Attempts were made to overcome this deficiency via kinetically stable bicelles, such as those comprising a mixture of the phospholipid 1,2-dipalmitoyl-sn-glycero-3-phosphatidylcholine (DPPC) and a sodium cholate-derived surfactant (SC-C5) at room temperature. These bicelles’ stability results from the high melting temperature of DPPC (41 °C) and a very low SC-C5 CMC (<0.5 mM) [158].

#### 2.2.2. Applications of Bicelles in Solubilizing and Stabilizing Integral Membrane Proteins

Typically, IMPs expressed in host membranes are first extracted and solubilized in detergents and then reconstituted in bicelles. Two basic protocols exist for reconstituting an IMP into bicelles: formulating the bicelles via the addition of detergent to proteoliposomes or integrating a detergent-stabilized IMP into bicelles [159,160] (Figure 3B). In addition, some studies on synthesized and usually truncated IMPs or on other membrane-associated protein constructs have used bicelles for direct solubilization. These hydrophobic proteins and protein constructs are first dissolved in an organic co-solvent, such as chloroform or TFE, and then mixed with the lipids before being lyophilized and dissolved in an appropriate buffer to form bicelles [161].

#### 2.2.3. Applications of Bicelles in Studies on Integral Membrane Proteins Using Biophysical and Structural Biology Methods

Small isotropic bicelles have been a highly preferred membrane mimetic platform in studies of IMP structure and dynamics by solution NMR spectroscopy, since they provide both a close-to-native lipid environment and fast enough tumbling to average out anisotropic effects, yielding good quality NMR spectra [146,160,162]. Still, IMP size is a serious limitation for solution NMR; and the need to produce isotopically labeled IMPs, given that their expression levels are typically small, introduces additional difficulty [36,151]. Nevertheless, the structures of several bicelle-reconstituted relatively large IMPs, such as sensory rhodopsin II [163], EmrE dimer [164], and the transmembrane domain of the receptor tyrosine kinase ephA1 [165], have been solved using solution NMR. Large bicelles have been the choice of solid-state NMR studies because they provide a greater bilayer surface and structural stabilization of the embedded IMPs. Beside the fact that large IMPs can be incorporated, the orientation of large bicelles in the external magnetic field can be controlled. Such bicelles can also be spun at the magic angle, enhancing spectral resolution for the embedded IMPs [151,166,167].

X-ray crystallography has also utilized bicelles to determine the high-resolution structure of IMPs in their native lipid environment, particularly in cases when detergents could not stabilize the IMP structure for crystallization [168]. Bicelle–IMP complexes can be handled similarly to detergent–IMPs and are compatible even with high-throughput robot-aided crystallization [169]. Thus, after the first successful crystallization of bicelle-residing bacteriorhodopsin [170], the crystal structures of several other IMPs, such as β2-adrenergic G-protein coupled receptor-FAB complex [171], rhomboid protease [172], and VDAC-1 [173] were solved.

Studies using EPR spectroscopy, pulse, and CW with spin labeling have also used bicelles as a lipid mimetic to study the conformational dynamics of IMPs. Magnetically aligned bicelles were used to probe the topology and orientation of the second transmembrane domain (M2δ) of the acetylcholine receptor using spin labeling and CW EPR [174]. Further, the immersion depth of the spin-labeled M2δ peptide at different positions in bicelles was determined. Here, CW EPR was used to monitor the decrease in nitroxide spin label spectrum intensity due to nitroxide radical reduction upon the addition of ascorbic acid [175]. Pulse EPR distance measurements on spin-labeled McjD membrane transporter in bicelles revealed functionally relevant conformational transitions [176].

### 2.3. Nanodiscs in Studies of Integral Membrane Proteins

#### 2.3.1. General Properties of Nanodiscs

**Figure 4:**
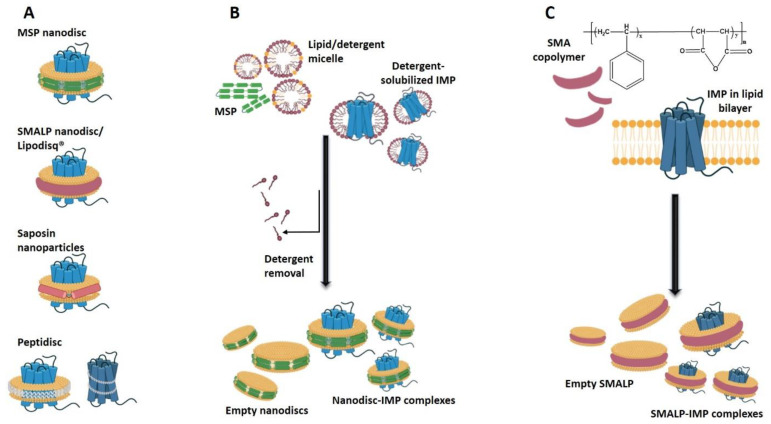
IMPs in nanodiscs. (**A**) IMP-nanodisc complexes of different types are shown. These are discoidal structures containing a segment of lipid bilayer with incorporated IMP surrounded by a belt of different nature that stabilizes the nanoparticle. Depending on the belt used, nanodisc can be IMP–MSP nanodisc, IMP–SMALP/Lipodisq^®^, IMP–Saposin nanoparticles, and IMP–peptidiscs with and without lipids incorporated. The size of nanodiscs can be controlled by changing the belt length to accommodate just one monomeric IMP or IMP oligomeric complex. (**B**) Typically, the detergent solubilized IMPs are transferred in nanodiscs by mixing IMP in detergent, MSP, detergent-solubilized lipids or mixed detergent–lipid micelles, incubated and the detergents are removed, in most of the cases by using BioBeads. As a result, IMP–nanodisc complexes and empty nanodiscs are formed. The empty nanodiscs can be removed further. (**C**) The IMP–SMALP/Lipodisq^®^ complexes can be formed by mixing CMA copolymer with liposome- or native membrane-residing IMPs. This is an advantage of using CMA copolymers, since they do not require the detergent-solubilization of lipid bilayer prior to IMP reconstitution, and can extract IMPs from the native membranes of expression host.

Sligar and colleagues were first to illustrate nanodisc technology in 1998 in a study focused on liver microsomal NADPH-cytochrome reductase enzyme, the CYP450 reductase [177,178]. The first nanodiscs were proteolipid systems made of lipid bilayer fragments surrounded by high-density lipoprotein (HDL). Thereafter, the diversity of nanodiscs expanded to include lipid nanostructures held intact by a belt of lipoprotein (membrane scaffold protein, MSP) [179,180], saposin [181], peptide [182], or copolymer [183]. All these membrane mimetics are self-assembled, nano-sized, and generally disc-shaped lipid bilayer structures (Figure 4). A major advantage of the nanodisc technology is the absence of detergent molecules and the ability to maintain integrity and shape upon dilution. This overcomes the shortcomings of lipid bicelles and provides a more native-like membrane environment compared to detergents [184,185]. Other advantages of nanodiscs are good accessibility of soluble domains in IMPs, sample homogeneity, and isolation of defined IMP oligomeric states by controlling the size of the nanodisc [186].

Currently, nanodisc systems are classified based mostly on the belt used. The most common type is *MSP nanodiscs* made by using the repeat domain of apolipoprotein A1 (ApoA1), the main component of DHL, which is referred to as membrane scaffold protein (MSP) [177] (Figure 4A). The formation of these nanodiscs requires two copies of the amphipathic α-helical MSP, which wraps up and stabilizes a small disc of lipid bilayer [151,177]. Both copies of MSP are arranged antiparallel to each other [187]. The size of nanodiscs can be controlled by using one or more MSP repeat regions, which are produced by protein engineering. For example, MSP1 consists of one repeat of 10 helices and MSP2 consists of two equivalent repeats each consisting of 10 helices [188,189]. Further modification in just one repeat, e.g., adding identical helices produced longer than the MSP1 constructs MSP1E1, MSP1E2, and MSP1E3, or deletions in MSP1 produced shorter constructs denoted MSLP1D1 and MSP1D2 [189]. Thus, any variation in the number of these amphipathic helical repeats results in different nanodisc diameters/sizes. For an empty nanodisc (one with no IMP incorporated), the type of phospholipid and the MSP construct establish the number of phospholipids in each particle, typically ~20 to 400 [184,188,189]. Sligar and colleagues [188,190] suggested the following correlation between the number of lipid molecules in the nanodiscs (*N_L_*) and amino acids in the scaffold protein (*M*):

(2) $$N_{L}\cdot S={\left(0.423\cdot M-9.75\right)}^{2}$$

where *S* represents the mean surface area per lipid used to form the nanodisc, measured in Å^2^.

The prototypical MSP1 construct forms nanodiscs with diameters of about 10 nm and has an overall molecular mass of approximately 150 kDa [188], but the modified MSP1 and MSP2 constructs can form smaller or larger nanodiscs with diameters ranging from about 8.4 nm to 17 nm [184,189]. Recently, nanodiscs with covalently linked N and C termini of newly engineered variants based on ApoA1 were developed, and termed covalently circularized nanodiscs (cNDs) [191].

*Copolymer nanodiscs* were introduced by Knowles and colleagues [192], who purified an IMP in polymer nanodiscs, i.e., Styrene–maleic acid–lipid particles (SMALPs). These nanodiscs were termed Lipodisq^®^ and are discoidal structures comprising of a segment of lipid bilayer surrounded by a polymer belt [193]. This belt is made of a styrene-maleic acid (SMA) copolymer formed by the hydrolysis of styrene-maleic anhydride (SMAnh) precursor and composed of 1:2 or 1:3 ratios of maleic acid to styrene [192]. The main distinction between MSPs and Lipodisqs is that SMA copolymer can directly cut out patches from the lipid bilayer without the use of detergents [192]. The principle of SMA-bound particles is centered on the interaction of the hydrophobic edge of a planar bilayer membrane with the styrene phenyl rings of the SMA polymer. This interaction stabilizes the disc-shaped SMALPs [69]. Monodisperse lipid discs with 140 lipid molecules and 10–11-nm diameter are formed with the help of SMA for the isolation of target membrane protein [194]. Lipodisqs with different incorporated lipids, e.g., palmitoyl-oleoyl-phosphocholine (POPC) [195] or DMPC [196], have been prepared and used. A major consideration when working with Lipodisqs is their pH-dependent stability, as they precipitate at pH values below 6.5 due to maleic acid moiety protonation, which is a disadvantage when studying IMPs at lower pHs. SMA polymer chelates divalent cations (e.g., Mg^2+^ and Ca^2+^) that are used for signaling assays, leading to Lipodisqs’ insolubility. To overcome these deficiencies, chemical modifications of maleimide carboxylates of SMA polymers with positively charged quaternary ammonium compounds (SMA-QA) or ethanolamine have been employed [197,198]. Another copolymer called DIBMA (di-isobutylene/maleic acid) was also developed—it is less harsh than SMA, stable in the presence of divalent cations owing to the absence of aromatic moiety, and does not interfere with far-UV optical spectroscopy [199].

*Synthetic peptide-based nanodiscs* (also termed “peptidiscs”) are formed by short amphipathic peptides aligned in an antiparallel fashion around the hydrophobic rim of a phospholipid membrane [182,200,201]. Bi-helical peptides displace detergent molecules by wrapping around the hydrophobic parts of detergent-purified membrane proteins [148,182]. Another example is a peptide derived from the ApoA1, which consists of 18 amino acids that form a single alpha helix of almost the same length as that of the apolipoprotein A1 helix [200,202,203]. Among the major benefits of peptidiscs is that their size can be adjusted by a simple variation in the peptide-to-lipid ratio. Also, peptide nanodiscs encapsulate IMPs irrespective of initial lipid content, so there is no need to consume exogenous lipids to match the diameter of the scaffold membrane as in the case of MSP nanodiscs. Furthermore, peptide stoichiometry is self-determined because the size and shape of the integrated IMP guide the binding of the peptide skeleton [69,204,205]. However, the comparatively high cost of custom peptide synthesis and its low stability due to their noncovalent assembly compared to the stability of other types of nanodisc systems are among the cons of the peptide nanodisc system [69,206].

*Saposin nanoparticles* are protein-stabilized lipid structures utilizing Saposin lipoprotein variants [207]. Salipro^®^, a Saposin A (SapA) disc, is the most suitable approach for IMP studies, since it can tolerate a wide range of lipid-to-Saposin ratios [208]. Salipro nanodiscs are composed of two or more SapA proteins that are joined together and assembled in V shapes around a small lipid disc, which makes them relatively flexible/tunable to accommodate different sizes of IMPs [181,209].

#### 2.3.2. Applications of Nanodiscs in Integral Membrane Protein Solubilization and Stabilization

Typically, detergent-solubilized IMPs are reconstituted into nanodiscs of different types, starting either from a whole solubilized membrane or after purification. Currently, the most widely used procedure is to transfer the purified detergent-solubilized IMP into nanodiscs—This is done by mixing the IMP, lipid and scaffold protein or polymer; thereafter, the detergent is removed using BioBeads and the nanodiscs with or without incorporated IMP are formed [190] (Figure 4B). Optimization to determine the optimum scaffold protein, polymer, or peptide, as well as lipid concentration to accommodate each particular IMP in its native oligomeric state, must be performed [186,210]. Procedures for the direct transfer of IMPs from the membrane into nanodiscs with minimal involvement of detergent have been utilized [211]. Lipodisqs have also been used to purify IMPs in native host membranes without any detergent, preserving the IMPs’ native state intolerance to detergents and preferences for particular lipids or lipid bilayers [53,212,213]. Furthermore, some advantageous technologies for cell-free expression of IMPs utilize direct incorporation and folding of the synthesized proteins into nanodiscs, which also benefits from the opportunity to tune the nanodiscs’ lipid composition [214,215,216].

#### 2.3.3. Applications of Nanodiscs in Functional Studies of Integral Membrane Proteins

As discussed above, one significant advantage of nanodiscs is that the soluble domains of IMPs reconstituted in them are well accessible. Therefore, binding of ligands, e.g., substrates, inhibitors, etc., and protein partners—all relevant to the IMP function—can easily be studied in a native-like environment. Thus, fluorescence correlation spectroscopy was used to assay fluorescently labeled IMPs’ binding interactions via an autocorrelation function, which depends on the diffusion coefficients of the bound vs. unbound species [217,218]. Scintillation proximity assay was used to assess radio–ligand binding to membrane transporters residing in nanodiscs, overcoming the protein activity reduction caused by detergents [219]. An assay measuring ATP hydrolysis by MsbA transporter in nanodiscs demonstrated the importance of MsbA–lipid interactions by varying the nanodisc lipid composition [220]. It was also found that nanodiscs facilitate the identification of monoclonal antibodies targeting multi-pass IMPs, which is important for antibody-based pharmaceutical developments [221].

#### 2.3.4. Applications of Nanodiscs in Studies of Integral Membrane Proteins Using Biophysical and Structural Biology Methods

Since their initial development, nanodiscs have been widely used in studies of IMPs’ structure and conformational dynamics due to their suitability to a variety of techniques and methods. As yet, crystallization of IMPs in nanodiscs for X-ray structure determination has proven a difficult task. However, crystallization of IMPs can be assisted by transferring them from nanodiscs/Lipodisqs to lipidic cubic phases (LCPs); high quality crystals of bacteriorhodopsin and rhodopsin crystals were obtained and the structures of these proteins solved at and below 2 Å resolution [17,221].

On the other hand, EM has greatly benefited from nanodiscs, and the first EM studies were on negatively stained nanodisc-IMPs, such as the dimeric bc_1_ complex and reaction centers from antenna-free membranes [222,223]. However, the structural resolution achieved was insufficient. Further technical developments in single-particle cryoEM have since made it possible to determine the high-resolution structure of IMPs in native lipid environments, capturing multiple functional protein conformations and oligomeric states [224,225]. Still, only proteins with sufficient molecular weight, typically about or above 150 kDa, can be visualized by the available advanced EM approaches and data processing. Thus, the structure of the ca. 320 kDa trimeric bacterial multidrug efflux transporter AcrB was resolved at a resolution of 3.2 Å in Lipodisqs, uncovering a well-organized lipid-bilayer structure associated with the protein transmembrane domain [226]. Also, the structure of nanodisc-embedded full-length glycine receptor at 3 to 3.5 Å resolution was resolved in the ligand-free, glycine-bound, and allosteric modulator-bound states, providing a comprehensive map of the functionally relevant conformational isomerizations [227]. CryoEM on SthK, a prokaryotic cyclic nucleotide-gated channel, also yielded high-resolution structures of channel apo, cAMP-bound, and cGMP-bound states in nanodiscs [228]. Remarkably, the structures of small IMPs were also resolved by EM in nanodiscs [229]. However, in these studies engineering of fusion protein or antibody/antigen-binding fragment (Fab) was utilized to increase the protein size and stability and succeed in the structure determination. For instance, the structure of 49 kDa *P. falciparum* CQ-resistance transporter PfCRT in complex with Fab was resolved at 3.2 Å resolution [230]. Consequently, nanodisc technology greatly improved the likelihood of understanding the structure of functionally relevant IMP conformations and visualizing essential protein–lipid interactions.

Nanodiscs have been particularly useful in studies of IMPs using NMR spectroscopy as well. Solution NMR has benefited from the fast tumbling of the nanodisc–IMP complex providing correlation times in the nanosecond range [34]. Still, the limitation of IMP size persists. Careful optimization of several parameters must be performed to obtain homogeneous samples with desired size: the scaffold protein/copolymer-to-lipid molar ratio; lipid composition, to provide hydrophobic match to the transmembrane part of IMP and/or specific interactions; and optimizations of nanodisc-to-IMP molar ratios [148,231]. This is true to an extent for all other structural biology techniques utilizing nanodiscs. Also, for solution NMR, reduced-size nanodiscs of 60–120 kDa with faster tumbling are more appropriate to obtain good NMR data quality [38,184]. Solid-state NMR studies have been conducted on complexes oriented in external magnetic field nanodisc/Lipodisq–IMP without magic angle spinning and on isotropic nanodisc/Lipodisq–IMP complexes with magic angle spinning [232]. Such studies open the opportunity to elucidate the high-resolution structure and conformational dynamics of IMPs in native-like environments. Nanodiscs have been useful in NMR applied to GPCRs and other physiologically and biomedically important IMPs [233,234].

EPR spectroscopy studies of spin-labeled IMPs’ structure–function relationships and conformational dynamics have also utilized nanodiscs as a membrane-mimetic platform [30,123]. Thus, double electron–electron resonance distance (DEER) measurements were conducted on a nanodisc-incorporated LmrP eukaryotic multidrug transporter [235]. In this study, the lipid makeup of the nanodiscs greatly affected the functional conformational state of the transporter. Lipodisq nanoparticles were used to assess the conformational dynamics of the human KCNQ1 voltage sensing domain [236]: The powerful combination of CW EPR and DEER confirmed the stabilization effect of the Lipodisqs on protein structure. In this study, the superior DEER data quality compared to liposomes highlighted the high potential of these membrane mimetics in studies of IMPs. The Aer primary energy sensor for motility in *E. coli* was also reconstituted in nanodiscs and studied by EPR [237]; although the DEER distances between the protein’s native Flavin radicals were very similar in detergent (DDM) and nanodisc environments, the observed protein activity was indeed higher in nanodiscs.

Nanodiscs were used in studies of IMPs by fluorescence-based techniques: internal reflection fluorescence microscopy (TIRFM), fluorescence correlation spectroscopy (FCS), and FRET were all applied to nanodisc-reconstituted cytochrome P450 3A4 and possible mechanisms for protein allosteric regulation were proposed [238,239]. Lipodisq-reconstituted KirBac1.1 potassium channels were studied by using smFRET to probe the structural changes that occur in this multimeric channel upon activation and inhibition [240]. IMPs in native nanodiscs, i.e., copolymer-solubilized native membranes, have also been studied using FRET [241].

### 2.4. Liposomes in Studies of Integral Membrane Proteins

#### 2.4.1. General Properties of Liposomes

**Figure 5:**
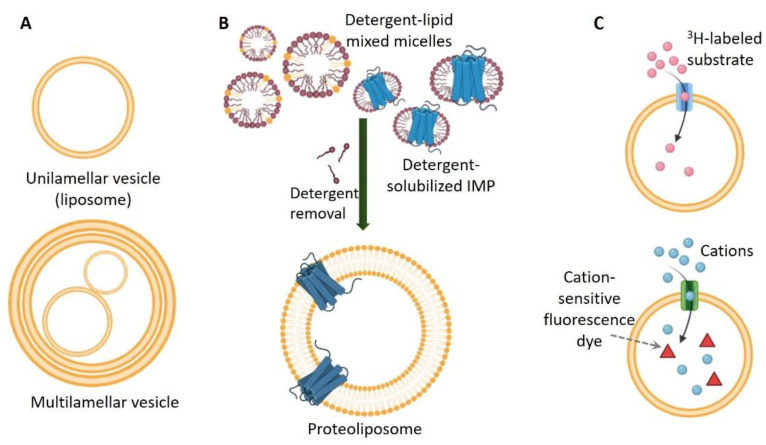
IMPs in liposomes. (**A**) Unilamellar and multilamellar vesicles are shown. These are continuous lipid bilayer structures with incorporated inside water-based solution, so the environment inside and outside of liposomes can be controlled. Typically, the unilamellar liposomes have just one lipid bilayer, whereas multilamellar vesicles have multi-bilayer onion-like structure with solution-filled compartments between the bilayers. (**B**) Typically, the IMPs are reconstituted in liposomes from detergent-solubilized form, which are mixed with detergent-solubilized lipids in the form of mixed detergent-lipid micelles. After some period of incubation to ensure IMP–lipid interactions, the detergent(s) are removed and the proteoliposomes, which usually have close to unilamellar morphology are formed. (**C**) The substrate uptake assay can be carried on liposome-reconstituted membrane transporters or channels: The uptake of radioactively (^3^H)-labeled substrate in the liposome can be quantified reporting on the IMP activity (upper panel) or the uptake of ions (cations) can be quantified by the changes in the fluorescence intensity of the liposome-incorporated dye, which is sensitive to the presence of substrate (lower panel).

Liposomes were introduced in 1961 by Bangham et al. [242] They are nano- and micro-sized vesicles that can have just one (unilamellar) or multiple (multilamellar) lipid bilayers [243,244] (Figure 5A). Unilamellar vesicles can range in size from 20 nm to more than 1 µM, and depending on their size are classified as small (20–100 nm), large (larger than 100 nm), or giant (larger than 1 µM), with the latter vesicles being closer to the size of a cell. Multilamellar vesicles have multilayer morphology and are greater than 500 nm in diameter. The inside lumen and the space between the lipid bilayers of the unilamellar and multilamellar vesicles are filled with water-based solution, and liposomes present a good artificial mimetic of a cell. Liposomes can be prepared from synthetic bilayer-forming phospholipids, but native membrane-extracted lipids have also been used [245]. Further, the physical and chemical properties of the lipid bilayer in liposomes can be tuned by varying the types and concentrations of lipids, and the amount of cholesterol added [246]. Generally, extrusion through polycarbonate filters can be used to prepare large unilamellar vesicles (LUVs) with a diameter of about 100–500 nm. Low-power bath sonication of lipid suspensions spontaneously forms small unilamellar vesicles (SUVs) with a diameter of about 20–50 nm. Hydrated phospholipids can be used to prepare giant unilamellar vesicles (GUVs) with a diameter greater than 500 nm by applying low-frequency electric fields. Other methods to produce liposomes include freeze-thawing and detergent extraction; hydration of lipid powders or films resulting inthe spontaneous formation of multilamellar vesicles (MLVs) with an overall size between 1 and 10 µm, as well [151,247,248,249]. Based on their properties that closely mimic biological membranes, liposomes have been extensively used in drug delivery due to their nontoxic nature and ability to encapsulate both hydrophilic and hydrophobic compounds [243,246,250,251].

Liposomes are also a great platform to reconstitute and study membrane proteins [248,252,253]. To this end, liposomes offer several unique advantages compared to other membrane mimetic systems. To begin with, multicomponent systems such as lipid, protein, and substrate complexes can be reconstituted in the liposomes because of the large size of this system [254]. Furthermore, liposomes sustain membrane potential because their hydrophobic bilayer introduces compartments in the aqueous phase, just like the native cells. In addition, liposomes represent a continuous membrane because they are not constrained by a solubilizing scaffold structure. This stands in contrast to other membrane mimetics, which only approximate a membrane bilayer. The diffusion behavior and native lateral pressure of phospholipids and proteins can be studied because of the continuous nature of liposome membranes [255]. All of these properties and the broad range of possible lipid compositions make these membrane mimetics an important tool to study IMPs’ conformational dynamics, substrate relocation across the membrane, folding, etc. at the molecular level [28,29,132,256,257,258]. In addition to liposomes, vesicles with similar properties termed “polymersomes”, which are made of amphiphilic polymers, have also been utilized in studies of biological processes at the membrane, or in drug delivery [259]. However, despite their high potential as membrane mimetics, the current applications of these membrane mimetics in IMPs structure-function studies are fewer compared to phospholipid liposomes, and therefore, their detailed description is beyond the scope of this review.

#### 2.4.2. Reconstitution of Integral Membrane Proteins in Liposomes

Typically, IMPs are transferred in liposomes from a detergent-solubilized state (Figure 5B). First, the desired lipids or lipid mixtures are transferred into a glass vial and dissolved in organic solvent. Then, the solvent is evaporated under a stream of nitrogen or argon gas and then under vacuum to remove the organic solvent completely; the preferred buffer for downstream experiments is added to the dry lipid film, and the lipids are hydrated for approximately 1 h at room temperature or 4 °C. depending on the lipid polycarbon chain saturation and temperature stability, vortexing or sonication can be applied as well. After complete lipid hydration, multilamellar vesicles are formed. Next, aliquots of the lipid suspension are taken in amounts needed to produce the desired final lipid-to-protein molar or *w*/*w* ratios and solubilized in mild detergent, e.g., Triton x-100. The detergent-solubilized IMP is mixed with the detergent-solubilized lipids and incubated for approximately 1 h at room temperature or a different temperature, if required. Finally, the detergents are removed to form proteoliposomes [28,29,132,249]. In the last step, the detergent can be removed by either dialysis or by using BioBeads. Also, further freeze–thawing, extrusion, or mild sonication can be performed to obtain more homogeneous and unilamellar proteoliposomes. It must be noted that the described method for IMP reconstitution in liposomes is rather challenging and requires optimization for each particular IMP. Currently, the most widely used method to obtain GUVs is electroformation [260]. This method has been utilized to incorporate IMPs as well—for example, the reconstitution of sarcoplasmic reticulum Ca^2+^-ATPase and H^+^ pump bacteriorhodopsin GUVs preserved these proteins’ activity [261]. Recently, a method to reconstitute an IMP into liposomes using native lipid binding without detergent solubilization was illustrated [248]. To do so, cytochrome c oxidase (CytcO) was first solubilized and purified in SMA nanodiscs (Lipodisqs) and then the protein–nanodisc complexes were fused with preformed liposomes, a methodology previously used for IMP delivery and integration into planar lipid membranes [262].

#### 2.4.3. Applications of Liposomes in Functional Studies of Integral Membrane Proteins

As noted above, proteoliposomes (IMP–liposome complexes) are similar to isolated cells to a certain extent: distinct environments of compounds, ions, or pH can be created inside and outside of liposomes, and in addition transmembrane potential can be generated [263,264,265,266,267]. This is a great advantage for the design and implementation of in vitro functional assays of IMPs. Typically, in these assays, the IMP liposomes, also known as unilamellar vesicles, are filled with the desired buffer, with or without IMP ligands, and aliquots of these proteoliposomes are then transferred to a bath buffer with significantly greater volume than that inside of the liposome. Thus, the reconstituted IMPs sense the difference between the buffers inside and outside the liposome. Such experimental setups are used, for example, to quantify the uptake of substrates by membrane transporters or channels, if the bath buffer contains a labeled substrate, e.g., radioactively labeled substrate [28,268,269], or the proteoliposomes are prefilled with a fluorescent dye whose intensity depends on the presence of substrate [270,271,272] (Figure 5C). In such experiments, the uptake of radioactive ^86^Rb into liposomes was utilized to measure the activity of channels reconstituted in these liposomes [268]. Radioactively labeled substrates (typically ^3^H-labeled, but other radioactive atoms can be used as well) have been widely used in liposome-based functional studies of membrane transporters, e.g., Na^+^-dependent dicarboxylate transporter [273] and Na^+^-dependent aspartate transporter GltPh [274]. A fluorescence-based method using Magnesium Green, a Mg^2+^-sensitive dye, was used to evaluate ATP/ADP exchange via mitochondrial adenine nucleotide translocase [271]. In a similar assay, either Ca^2+^- or Na^+^-sensitive fluorescent probes entrapped in liposomes containing connexin 26 hemichannels were used to demonstrate for the first time the translocation of Ca^2+^ by the connexin channel [270]. Inhibitors of IMPs have also been tested in liposome-based assays [263]. Using different lipid mixtures to prepare liposomes was also exploited to study specific IMP–lipid interactions. Thus, the activity of mammalian glucose transporter depends upon anionic (phosphatidic acid, phosphatidylserine, phosphatidylglycerol, and phosphatidylinositol) and conical phospholipids (phosphatidylethanolamine and diacylglycerol) [265].

#### 2.4.4. Applications of Liposomes in Studies of Integral Membrane Proteins Using Biophysical and Structural Biology Methods

Due to their complexity, attempting to determine the high-resolution structure of IMPs in proteoliposomes is usually not a researcher’s first choice. Still, liposomes have been used to crystallize IMPs incorporated in the bilayer, and the obtained 2D crystals were analyzed by EM [258,275]. Although using EM to characterize the structure of IMPs from 2D crystals formed in flattened liposomes is a difficult task due to varying liposome morphology and other factors, success was achieved. Electron cryotomography, subtomogram averaging, and electron crystallographic image processing were successfully applied to analyze the structure of bovine F1Fo ATP synthase in 2D membrane crystals [276]. Another advancement in determining the structure of IMPs using 2D crystallization of liposomes is to generate buffer gradient from the inside to the outside of the liposome, which activates the IMP. Then, the 2D crystals are quickly frozen under liposome gradient conditions and snapshots of active protein are taken. This technique has contributed to the detailed characterization of IMP functional conformations in lipid bilayers [258].

Conformational dynamics underlying IMPs’ function in liposomes have been extensively studied using EPR spectroscopy [27,28,29,30,32,119,132]. This technique can be applied to IMPs in both unilamellar and multilamellar vesicles and is not restricted based on the size of proteins in the liposome. In many cases, EPR studies were conducted on the same proteins in detergent and in liposome, revealing distinct membrane-mimetic dependent conformational behavior. Using DEER spectroscopy for the GltPh transporter, Georgieva et al. [28] found that although the subunits in this homotrimeric protein occupy the outward- and inward-facing conformations independently, the population of protomers in an outward-facing state increases for proteins in liposomes. Also, the lipid bilayer affects the assembly of the M2 proton channel from influenza A virus as deduced from DEER modulation depth measurements on spin-labeled M2 transmembrane domain in MLVs compared to detergent (β-DDM)—the dissociation constant (K_d_) of M2 tetramer is significantly smaller than that in detergent, therefore the lipid bilayer environment facilitates M2 functional channel formation [29,132]. These studies are extremely important in elucidating the role of lipid bilayers in sculpting and stabilizing the functional states of IMPs.

Single-molecule fluorescence spectroscopy and microscopy have also been used to study conformations of IMPs in liposomes. This technique was used to successfully assess the dimerization of fluorescently labeled IMPs [277,278] and the conformational dynamics of membrane transporters in real time [137,279].

### 2.5. Other Membrane Mimetics in Studies of Integral Membrane Proteins

#### 2.5.1. Amphipols

The concept of amphipols—amphipathic polymers that can solubilize and stabilize IMPs in their native state without the need for detergent—emerged in 1994. Amphipols’ mechanism was validated in a study of four IMPs: bacteriorhodopsin, a bacterial photosynthetic reaction center, cytochrome b6f, and matrix porin [280]. Amphipols were developed to facilitate studies of membrane proteins in an aqueous environment by providing enhanced protein stability compared to that of detergent [281,282]. Functionalized amphipols can be used to trap membrane proteins after purification in detergent, during cell-free synthesis, or during folding [281]. Because of their mild nature, amphipols provide an excellent environment for refolding denatured IMPs, like those produced as inclusion bodies [283]. The stability of IMP–amphipol complexes upon dilution in an aqueous environment is another advantage of these membrane mimetics. Thus, amphipols have been used in numerous IMP studies to monitor the binding of ligands and/or determine structures [280,284]. Still, they have some disadvantages. Their solubility can be affected by changes in pH and the addition of multivalent cations, which neutralize their intrinsic negative charge and lead to low solubility [284,285].

#### 2.5.2. Lipid Cubic Phases

Lipidic cubic phase (LCP) is a liquid crystalline phase that forms spontaneously upon mixing of lipids and water under specific conditions [286,287]. It was introduced as membrane mimetic in 1996 for crystallization of IMPs [18]. Since then, numerous IMP structures that had been difficult or even impossible to crystalize in other mimetic environments were solved in LPC [19,288]. The first structure of GPCR as a fusion construct with T4 lysozyme was solved in LPC by Kobilka et al. [289] LCP can be described as highly curved continuous lipid bilayer made of monoacylglycerol (MAG) lipids, which is surrounded by water-based mesophase. Thus, the whole system forms continuous highly curved channels, in which IMPs are incorporated. Generally, LCPs maintain the IMPs functional conformations and activity. For crystallization in LCPs, the detergent-solubilized IMP is mixed with the LCP-forming lipid, to which specific lipids can be added as well. The addition of precipitant to this system affects the LCP in terms of phases transition and separation, so some of these phases become enriched in IMP leading to nucleation and 3D crystals growth. In addition to crystallography, functional assays have been performed on LPC-reconstituted IMPs as well [290]. Due to space limitations, we do not provide further details of this highly advantageous for X-ray crystallography and protein structure determination. More details can be found in specialized reviews elsewhere [286,291].

## 3. Conclusions

Due to the important roles of IMPs in cells’ and organisms’ normal physiology as well as in diseases, there is a need to comprehensively understand the functional mechanisms of these proteins at the molecular level. To this end, in vitro studies on isolated proteins using diverse biochemical and biophysical approaches provide invaluable information. However, studies of IMPs are challenging due to these proteins’ hydrophobic nature, low expression levels in heterologous hosts, and low stability when transferred out of the native membrane to a membrane-mimetic platform. To overcome these challenges, progress has been made in multiple directions. We summarized the developments of lipid membrane mimetics in functional and structural studies of IMPs over the past several decades. Indeed, the diversity of these systems grew significantly, and the widely ranging lipid membrane-mimetic platforms now available provide high solubility, stability, more or less lipid-bilayer environments, and other specific properties that are utilized in studies featuring NMR, X-ray crystallography, EM, EPR, fluorescence spectroscopy assays, ligand binding and translocation assays, etc. This has resulted in the continuous expansion of knowledge about IMPs. In Table 1, we provide concise information about the most-widely used membrane mimetics to study IMPs, selected applicable techniques, along with some of their advantages and disadvantages.

The fast development of lipid membrane mimetics and the great expansion of their diversity also provides a great promise for the successful future research to uncover the mechanisms of IMPs, which, to date, have been difficult to stabilize and study. Besides, combining the information from studies of IMPs in different membrane mimetics and by different techniques will help to more completely understand the structure and function of these proteins and avoid possible biases due to the selection of membrane environment.

## Figures and Tables

**Table 1:** Summary of most widely used lipid membrane mimetics in functional and structural studies of IMPs.

**System/Type**	**Applicable Techniques to Study IMPs**	**Advantages**	**Disadvantages**
**Detergent micelles** Ionic detergents Zwitterionic detergents Non-ionic detergents	X-ray crystallography Single-particle cryoEM Solution NMR EPR spectroscopy Fluorescence spectroscopy smFRET Isothermal titration calorimetry (ITC) for ligand binding/protein interactions Functional assays	Easy handling Starting point for downstream applications Availability of large variety of detergents	Propensity of IMP denaturation Chances of non-physiological IMP conformations due to mismatched ‘IMP-micelle’ hydrophobic thicknesses CMC of the detergent must be considered
**Bicelles**	Solution NMR Solid-state NMR X-ray crystallography EPR spectroscopy	Easy preparation Homogeneous and translucent suspensions Provide true lipid environment physiological conditions Diverse types of lipids can be incorporated to match Bicelles of different sizes can be prepared	Total lipid concentration can affect size and geometry of bicelle Risk of IMP perturbation in case of insufficient bilayer size
**Nanodisc** MSP nanodiscs SMALP/Lipodisq^®^ Synthetic peptide-based nanodiscs Saposin nanoparticles	Single particle cryoEM Solution NMR Fluorescence spectroscopy and microscopy smFRET EPR spectroscopy ITC for ligand binding/protein interactions Functional assays	Maintain integrity and shape even upon dilution Easy accessibility of soluble domains in IMPs Possibility of size adjustment to accommodate a monomeric IMP or larger IMP complex	Optimization of assembly conditions can be time consuming Not suitable for large MP oligomers Dynamics of lipids affected by protein ‘belt’ Limited size range
**Liposomes** Small unilamellar vesicles (SUVs) Large unilamellar vesicles (LUVs) Giant unilamellar vesicles (GUVs) Multilamellar vesicles (MLVs)	Electron crystallography Solid-state NMR EPR spectroscopy smFRET Functional assays/substrate uptake Electrophysiology	Large size can accommodate large and multicomponent systems Represent continuous membrane providing closer to native environment for IMPs Diffusion behavior similar to native phospholipid membrane Broad range of possible lipid compositions	The orientation of IMP is often non-native Expensive compared to the traditional systems Low solubility
**Amphipols**	Single-particle cryoEM Solid-state NMR	Assist IMPs study in aqueous environment Stability of IMP-amphipol complex stable on dilution Provides better IMP stability compared to micelle Facilitate refolding of denatured IMPs	Commercially evaluability of only one amphipol type Too difficult to maintain the IMP-amphipol complex sometimes Multivalent cations- and pH-dependent solubility
**Lipidic cubic phase**	X-ray crystallography Functional studies	More native-like environment for IMPs facilitating their crystallization	Relatively expensive
